# Pharmacological Management of Neuropathic Pain after Radiotherapy in Head and Neck Cancer Patients: A Systematic Review

**DOI:** 10.3390/jcm11164877

**Published:** 2022-08-19

**Authors:** Maria Kouri, Martina Rekatsina, Athina Vadalouca, Ioanna Siafaka, Emmanouil Vardas, Erofili Papadopoulou, Antonella Paladini, Giustino Varrassi

**Affiliations:** 1 A’ Anesthesiology Clinic, Pain Management and Palliative Care Center, Aretaieio University Hospital, Medical School, National and Kapodistrian University of Athens, 11528 Athens, Greece; 2 Dental Oncology Unit, Department of Oral Medicine and Pathology and Hospital Dentistry, Dental School, National and Kapodistrian University of Athens, 11527 Athens, Greece; 3 Department of Anesthesia Pain Therapy and Palliative Care, Aretaieio University Hospital, Medical School, National and Kapodistrian University of Athens, 11528 Athens, Greece; 4 Pain Clinic, Athens Medical Center, 15125 Athens, Greece; 5 Department of MESVA, University of L’Aquila, 67100 L’Aquila, Italy; 6 Paolo Procacci Foundation, 00193 Roma, Italy

**Keywords:** head–neck cancer, neuropathic pain, radiotherapy, pharmacological therapy

## Abstract

Background: Neuropathic pain (NP) in head and neck cancer (HNC) patients represents a treatment challenge. Most studies investigating drugs against NP are conducted in patients suffering with diabetic neuropathy or postherpetic neuralgia, while data are limited in cancer pain management. Additionally, regarding cancer therapy-related NP, most of the studies do not focus on HNC patients. The aim of this review is to identify the studies on systematically administered medication for NP management that included HNC patients under radiotherapy. Methods: A systematic literature search was performed, following the Preferred Reporting Items for Systematic Reviews and Meta-Analyses (PRISMA) guidelines, in PubMed, Cochrane Library, Web of Science and ClinicalTrials.gov on 30 October 2021. The medical subject heading (MeSH) terms were (“head and neck cancer” OR “tumor”) AND “neuropathic pain” AND “medication” AND “radiotherapy.” The Cochrane Collaboration tool was used for quality assessment. Results: The search identified 432 articles. Three more articles were identified after searching the reference lists of the retrieved articles. A total of 10 articles met the eligibility inclusion criteria and were included in this review; 6 on gabapentin, 1 on pregabalin, 1 on nortriptyline, 1 on methadone, and 1 on ketamine. Statistically significant results in pain reduction compared to placebo or standard pain medication were found in the studies on pregabalin (*p* = 0.003), methadone (*p* = 0.03), ketamine (*p* = 0.012), and in two out of six gabapentin studies (*p* < 0.004). Two of the studies (both concerning gabapentin) had no comparison arm. Conclusions: Treatments including pregabalin, methadone, ketamine, and gabapentin were found to provide pain relief against HNC NP. While there is a plethora of pharmacological treatments available for the management of NP, only a few studies have been conducted regarding the pharmacological management of therapy-related NP in HNC patients. More studies should be conducted regarding the pharmacological approaches in HNC therapy-related NP so that specific treatment algorithms can be developed.

## 1. Introduction

Neuropathic pain (NP) is defined as “pain caused by a lesion or disease of the somatosensory nervous system” [1], including peripheral fibers (Aβ, Aδ, and C fibers) and central neurons. NP is often associated with tingling or numbness of the affected area and is described as “electric shocks” or “bee stings” or “pins and needles”. It can be sharp, shooting and/or burning [2]. NP negatively impacts quality of life, resulting in a great burden for patients as well as for the health care system as patients consume a high quantity of resources [3]. NP in head and neck cancer (HNC) patients often remains under-reported, underdiagnosed, and undertreated [3].

In HNC patients, pain may be nociceptive, but more frequently, it has one or more neuropathic components [4,5,6]. In most of the cases, it is caused by cancer therapy and may develop following radiotherapy (RT), targeted therapy, and/or immunotherapy [7,8,9]. Oral mucositis (OM) is a frequent complication of these therapies, typically manifesting as erythema or ulcerations [4]. Oral mucositis-induced pain (OMP) is described by HNC patients as one of the most devastating side effects of RT. It results in patient distress and suffering and may be responsible for the necessity to use feeding tubes, the administration of opioid medication, treatment interruptions, hospitalizations, and increased medical costs [10,11]. Even though the severity of OMP is often related to OM, due to mucosal atrophy and ulceration [11,12], some studies have not found such a relationship [4,13,14].

NP is a well-recognized problem in HNC patients [4,5]; treatment-induced polyneuropathy [15] and OMP are associated with NP [4,5,10,16]. Around 20% of HNC patients under RT develop NP [4,6]. The actual therapy-related neuron damage as well as persistent unrelieved pain induced by the ulcers of OM can exacerbate the dysfunction in the nervous system, producing NP. Moreover, chemical mediators such as substance P, reactive oxygen/nitrogen species, tumor necrosis factor alpha (TNF-α), and bradykinin are elevated in OM and contribute to NP generation [5,17].

NP represents a therapeutical challenge in cancer patients as they are already receiving several drugs [18]. Recognizing the coexistence of NP, along with other types of pain is mandatory [19]. Additionally, the accurate diagnosis and distinction of NP elements from other kinds of pain is fundamental for the correct management of patients since NP needs a different treatment pathway.

While there is a plethora of pharmacological treatments available for the management of neuropathic pain, only a few studies have been conducted regarding the management of NP in HNC patients under RT. The Neuropathic Pain Special Interest Group (NeuPSIG) of the IASP (International Association for the Study of Pain) [20], and more recently, other authors [21] have published systematic reviews on the use of drugs for neuropathic pain. The publications included anticonvulsant drugs, monoclonal antibodies, tricyclic antidepressants, serotonin and noradrenaline reuptake inhibitors (SNRIs), opioids, topical lidocaine, capsaicin high-concentration patches, and herbal therapies. Most of the studies were conducted in diabetic neuropathy or postherpetic neuralgia, and data were limited to noncancer pain in adults. Regarding cancer-related neuropathic pain, most of the reviews do not focus on HNC patients.

In this review, we tried to identify the systematically administered drugs related to NP relief in HNC patients under RT.

## 2. Methods

### 2.1. Literature Search Strategy

A systematic literature search, following the Preferred Reporting Items for Systematic Reviews and Meta-Analyses (PRISMA) guidelines [22], was performed in the following databases: PubMed, Cochrane Library, and the Web of Science on 30 October 2021. The medical subject heading (MeSH) terms used for PubMed and Cochrane Library were (“head and neck cancer” OR “tumor”) AND “neuropathic pain” AND “medication”. For the Web of Science, they were “head neck cancer” AND “neuropathic pain”. The search in PubMed was narrowed applying the filters: *Full text*, *Clinical Trial*, *Meta-Analysis*, *Randomized Controlled Trial*, *Review*, *Systematic Review*, *Humans*, and *English* and in the Web of Science, *Review Articles*, *Early Access*, *Open Access*, and *English.* In the Cochrane Library, the search was limited to *title*, *abstract*, and *key words*. ClinicalTrials.gov was also searched, where the “condition or disease” was “*head and neck cancer*,” the “other terms” was “*pain medication*”, and the filters applied were “*with results*” and “*completed*”. The reference lists of the articles that met the eligibility criteria were further screened to identify additional eligible studies.

### 2.2. Eligibility Criteria

Only studies involving human subjects and published in English were selected. The aim of this review was to identify studies including HNC patients treated with RT and investigating the management of NP. All articles reporting NP or NP management as outcomes, as well as the ones reporting the use of adjuvant medication known for the management of NP or neuropathy were included. Articles that did not include HNC patients, did not refer to NP or medication related to NP, were not original studies, and nonpharmacological studies were excluded.

After the removal of duplicates, all article titles and abstracts were screened by two authors (MK, EP). Any differences of opinion were resolved by a third author (AV). All articles appearing to be eligible were assessed again as full texts by two authors (MK, MR), and disagreements were settled by a third one (AV).

### 2.3. Quality assessment of Included Studies

The seven domains of the Cochrane Collaboration risk of bias framework [23] were assessed for each of the included studies. The validity of each study was assessed as at low, unclear, or high risk of bias. A low risk of bias was given when there was a low risk of bias for all key domains assessed, except for “other bias”. An unclear risk of bias was indicated when there was an unclear risk for one or more domains (except “other bias”), and a high risk of bias was assigned when there was a high risk of bias for one or more key domains. The GRADE approach [24] was used to rate the quality assessment of the included studies.

### 2.4. Process of Data Extraction

Following the identification of the eligible papers, the relevant data were extracted from each article. The included information were: study-related data (first author, study type, drug and control/comparison); study characteristics (sample size, cancer site, radiotherapy received, and dosage of the used drug against NP); and outcome-related data (timing of intervention, pain assessment, outcome, and adverse effects).

### 2.5. Presentation of the Results

In this review, the studies are descriptively presented, based on the used drug. Due to the significant heterogeneity, it was not possible to use a meta-analytical method. Thus, it was preferred to use the “vote counting” method to draw conclusions. This method is one of the simplest ways to combine the results of many studies [25]. Each study is recorded in one of the categories: positive effect, negative effect, and zero effect. The category that gets the highest score will give the tendency of the final result of the intervention effect in relation to the control. The method is not of high quality as it does not evaluate the completeness of the methodology of the studies. It is used as a descriptive method only if there are insufficient data to perform statistical tests. However, in this case, it was the only method that could provide the overall trend of the final results.

### 2.6. Compliance with Ethics Guidelines

As this article is based on data from previously published research and does not involve any human enrollment, ethics committee approval was not required.

## 3. Results

### 3.1. Search Results

**Figure 1:**
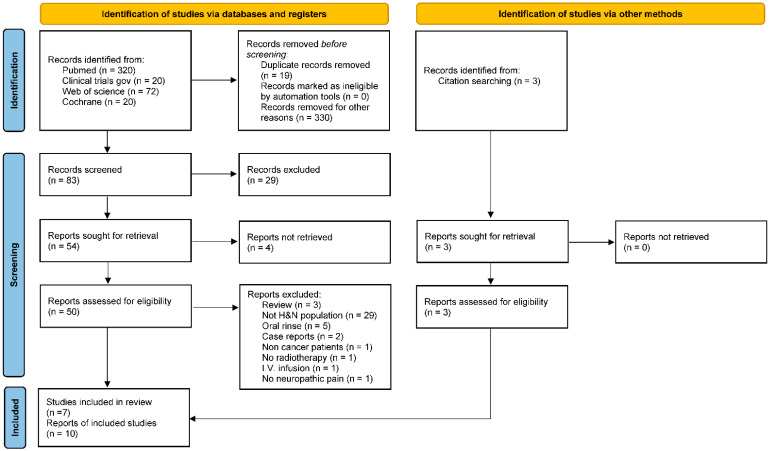
PRISMA 2020 flow diagram.

The research identified 432 articles. After duplicate removal, 413 of them were screened based on title and abstract, and 359 were removed because they were not consistent with the purpose of this review. Four articles were not retrieved as a full text. Forty-three articles were excluded after reading the full text; 29 of them because they did not concern HNC patients. Three articles were identified after searching the reference lists of the retrieved articles. A total of 10 articles were included in this review (Figure 1).

Out of the 10 articles included: 3 were prospective, randomized pilot studies [26,27,28]; 2 randomized, controlled trials [29,30]; 2 retrospective cohort studies [31,32]; 1 randomized trial [33]; 1 historically controlled study [34]; and 1 uncontrolled, open-label study [35]. Six of the studies were investigating gabapentin [26,27,28,31,32,34], one pregabalin [29], one nortriptyline [33], one methadone [30], and one ketamine [35]. Two of the studies did not have comparison arms [31,32]. The primary cancers were oral, oropharyngeal, laryngeal, hypopharyngeal, unknown primary, paranasal sinus, nasopharyngeal, tonsil, soft palate, thyroid, salivary glands, skin, paragangliomas, sino-nasal, lip, tongue base, and tongue (Table 1).

### 3.2. Quality Assessment of Included Studies

**Figure 2:**
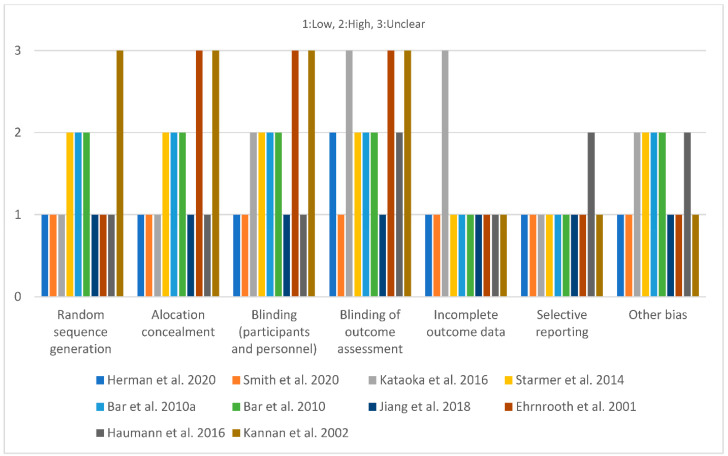
Quality Assessment of Included Studies. (Herman et al., 2020 [26]; Smith et al., 2020 [27]; Kataoka et al., 2016 [28]; Jiang et al., 2018 [29]; Haumann et al., 2016 [30]; Bar et al., 2010a [31]; Bar et al., 2010 [32]; Ehrnrooth et al., 2001 [33]; Starmer et al., 2014 [34]; Kannan et al., 2002 [35].

On the first domain “random sequence generation”, six of the ten studies had a low risk of bias [26,27,28,29,30,33]. In three, the risk was high [31,32,34], and in one, it was unclear [35]. For the domain “allocation concealment”, five studies had a low risk of bias [26,27,28,29,30]. Three had a high risk [31,32,34], and two had unclear risk of bias [33,35]. There was a high risk of bias for the domain “blinding of participants and personnel” in four studies [28,31,32,34], a low risk in four studies [26,27,29,30], and an unclear risk in two studies [33,35]. On the domain “blinding of outcome assessment”, two studies had a low risk [27,29], five had a high risk [26,30,31,32,34], and three had an unclear risk of bias [28,33,35]. In the next domain “incomplete outcome data”, there was a low risk of bias in nine studies [26,27,29,30,31,32,33,34,35], and an unclear risk of bias in one study [28]. On the last domain “selective reporting”, nine studies were judged to have a low risk of bias [26,27,28,29,31,32,33,34,35] and one to have an unclear risk of bias [30] (Figure 2). The lowest cumulative risk level of bias was found in two studies [27,29].

### 3.3. Pain Evaluation

Pain was assessed using various scales such as VAS (Visual Analog Scale), the Likert scale, NRS (Numeric Rating Scale), a 4-point scale (absent-0, mild-1, moderate 2-, severe-4) or opioids analgesia necessity. In the context of multidimensional, questionnaires such as OMWQ-HN (Oral Mucositis Weekly Questionnaire adapted for Head and Neck Cancer), EORTC QLQ-H&N35 (European Organization for Research and Treatment of Cancer Quality of Life Questionnaire—Head and Neck Module), VHNSSv2 (Vanderbilt Head and Neck Symptom Severity Survey version 2.0), BPI-SF (Brief Pain Inventory—Short Form), and the McGill PQ (McGill Pain Questionnaire) were used. OMWQ-HN and EORTC QLQ-H&N35 provide the responses of patients on domains related to mouth and throat soreness and pain. Neuropathic pain was assessed using DN4 (Douleur Neuropathique 4) as well as LANSSQ (the Leeds Assessment of Neuropathic Symptoms and Signs questionnaire) (Table 2).

The time points of pain assessment and recording varied between studies. It was either a fixed time point after the first radiotherapy session or when patients reported a specific pain score or if analgesia with used analgesics was not acceptable (Table 2).

### 3.4. Efficacy and Safety

The outcomes and the adverse effects of the drugs included in the review are summarized in Table 3.

### 3.5. Gabapentin

Six out of the ten included studies explored the efficacy of gabapentin in HNC patients treated with radiotherapy [26,27,28,31,32,34]. Two of them were prospective, randomized pilot studies [26,27], and one was an open label, prospective, randomized study [28]. Two were retrospective cohort studies [31,32] and one was a historically controlled study [34].

Hermann et al. [26] performed a prospective, randomized, pilot study in 60 patients; 31 received high-dose gabapentin (2700 mg daily) with either hydrocodone and/or paracetamol or fentanyl and/or paracetamol, if needed. Twenty-nine received low-dose gabapentin (900 mg daily) with methadone. Although there was no statistical difference in pain results between the treatment arms, more patients receiving high-dose prophylactic gabapentin did not require opioid administration during treatment (42% vs. 7%; *p* = 0.002).

Another prospective, randomized pilot trial included 79 patients [27]. Thirty-eight of them were randomized to usual care (mouthwash including lidocaine, nonsteroidal anti-inflammatories, and opioid analgesics) and 41 to usual care plus gabapentin, titrated up to 900 mg TID, continued throughout radiotherapy until analgesia was no longer required. Gabapentin administration resulted in lower pain levels (*p* = 0.004). By week 7, the median pain score in the gabapentin group was below the 0.25 quantile of the control group.

Kataoka et al. [28], in an open label, prospective, randomized study, compared the efficacy of gabapentin (up to 900 mg per day) plus standard pain control (SPC): paracetamol plus opioids in nine patients versus SPC alone in eleven patients. A nonsignificant difference was found in the median maximum VAS score in the gabapentin group compared to the SPC group (74 and 47, respectively; *p* = 0.552). In addition, there was no statistical difference between groups either in VAS scores from baseline to 4 weeks after the treatment, at each time point between two arms or in the number of days until the use of additional analgesics.

Starmer et al. [34] compared prophylactic use of gabapentin (2700 mg/d) with standard treatment (including opioid use) in a historically controlled study of 23 patients. The maximum pain scores recorded in the gabapentin group were significantly lower compared to the control group (*p* = 0.0003). Additionally, the gabapentin group had shorter overall pain duration compared to the controls (71.68 versus 239.55 days, *p* = 0.038). The 13% of the patients receiving gabapentin did not require additional analgesics, while, in the control group, all patients required opioids, and 70% of them required multiple opioid therapy.

A retrospective cohort study included 29 patients during radiotherapy treatment and receiving a median dose of gabapentin of 2700 mg per day at weeks 3, 4, 5, and 6 [31]. Only 10% (3/29) of the patients required additional doses of opioids at weeks 3 and 4. At weeks 5 and 6, 35% (10/29) of them required additional low doses of opioids.

In another retrospective cohort study on 42 patients, a median dose of gabapentin of 2700 mg per day at weeks 2, 3, 4, and the last week of radiotherapy was used [32]. The patients requiring an additional median dose of 10 mg/d of oxycodone equivalent were, respectively: at week 2, 12% (5/42); at week 3, 33% (14/42); and at week 4, 55% (23/42). At the last week of radiotherapy, 71% (30/42) of the patients required 60 mg/d of oxycodone equivalent.

A small number of patients experienced mild side effects in all studies, ranging from 5% to 33% of the patients. The described adverse events were dizziness, nausea, vomiting, follicular skin rash/allergic skin reaction, somnolence, vertigo, headache, drowsiness, and fatigue. In one of the studies [26], 3% of the patients discontinued treatment due to intolerance to gabapentin (one due to nausea and one due to difficulties swallowing liquids).

### 3.6. Pregabalin

A randomized, double-blind, placebo-controlled trial included 128 patients divided in two groups (pregabalin and placebo) [29]. Eligible patients had a mean pain intensity score of 4 or more on an 11-point NRS. At week 16, a decrease of 2.4 in pain intensity was found in the pregabalin group compared to 1.6 in the placebo group (*p* = 0.003). Around 30.0% (19/64) of patients receiving pregabalin compared to 7.8% (5/64) receiving the placebo achieved pain relief of 50% (*p* = 0.003). At the same time, there was also a significantly greater reduction of the mean Brief Pain Inventory interference total score in the pregabalin group (13.4) compared to the placebo group (8.6) (*p* = 0.001). Moreover, the proportion of patients receiving pregabalin and having a reduction in pain intensity of 30% or more at week 16 was also larger compared to the placebo group (59.4% (38/64) and 32.8% (21/64), respectively, *p* = 0.006).

In this study, patients experienced at least one adverse event in both the pregabalin and placebo groups (54.7% and 45.3%, respectively). Dizziness, somnolence, facial edema, increased pain, headache, diarrhea, and peripheral edema were described as adverse events. One patient in the pregabalin group discontinued treatment because of facial edema, and two patients in the placebo group discontinued the treatment because of hospitalization for increased pain.

### 3.7. Nortriptyline

This study compared nortriptyline (19 patients) to oral morphine (20 patients) in a randomized trial [33]. Patients in either treatment arm received supplementary medication from the opposite treatment arm if they had insufficient pain control. VAS scores in nortriptyline group were significantly higher 1 and 2 weeks after randomization compared to VAS scores in the morphine group (*p* = 0.007 and *p* = 0.04, respectively). No significant changes in pain were observed within groups from baseline to 1 and 2 weeks after randomization. The Likert pain scale scores showed a nonsignificant trend toward higher pain scores in the nortriptyline group compared to the morphine group at baseline and 1 week after randomization.

Nausea, vomiting, constipation, and CNS symptoms in 14 patients in each of the nortriptyline and control groups were reported as adverse events. In the nortriptyline group, cardiac arrhythmia was reported.

### 3.8. Methadone

A randomized, controlled trial was performed on 52 patients reporting pain scores NRS > 4 and DN4 > 4 [30]. The patients were naïve to strong opioids, and half of them received methadone, while the other half received fentanyl. A higher reduction in NRS was found at 1, 3, and 5 weeks in the methadone group (pain change 2.9, 3.1, and 3.1) compared to the fentanyl group (1.4, 1.7, and 2.0). A statistically significant difference was found at week 1 (*p* = 0.011) and 3 (*p* = 0.03). Improvement >50% at 1 week was higher in the methadone group compared to the fentanyl group (15% versus 50%, *p* = 0.012).

Xerostomia was the most common side effect in both groups (about 70% of the patients, at any point in the study), with no significant difference between the groups. Moreover, sleepiness, dizziness, nausea, vomiting, constipation, somnolence, and drowsiness were also reported. There were no serious side-effects or drop outs due to intolerable side effects.

This study has several limitations, e.g., the initial dosage of fentanyl was low (12.5 µg/h), and during the study, the dosage of methadone was changed from 2.5 mg *bis in die* (BID) to 2.0 mg BID for technical reasons. There are also no indications on the amount of rescue medication used by the two randomized groups. Moreover, the analgesia after 5 weeks was not significantly different.

### 3.9. Ketamine

Oral ketamine was evaluated as an adjuvant to oral morphine in cancer patients experiencing neuropathic pain [35]. Out of the nine cancer patients included in the study, four had HNC. All patients were reporting a NRS score ≥6 even though they were receiving maximally tolerated doses of either morphine, amitriptyline, sodium valproate, or a combination of these drugs for intractable neuropathic pain. Orally administrated ketamine in a dose of 0.5 mg/kg/body weight TID was used as an add-on therapy to the existing pharmacologic regimen. A decrease of more than three from the baseline in the average pain score, or a NRS ≤ 3 was taken as a successful response. All four HNC patients had a successful analgesia after ketamine.

Among the four HNC patients, three developed sedation, one vomiting, one anorexia, one nausea, three drowsiness, and one tiredness. The sedation score gradually improved despite continuing medication in all patients.

### 3.10. Results of the “Vote-Counting” Method

Table 4 summarizes the results of the “vote-counting” method. More specifically, of the 10 studies that were included in this review, 7 reported a positive effect [27,29,30,31,32,34,35] in the treated groups, and 3 reported a zero effect [26,28,33].

## 4. Discussion

This systematic review aimed to identify the successful analgesia in HNC patients under RT suffering with NP. It highlighted that despite the plethora of scientific publications on different pharmacologic therapies available for the management of NP [20,21], only a few of them are available for HNC patients receiving RT and experiencing NP. All data meeting the inclusion criteria for this systematic review derive from studies investigating the systemic administration of drugs. Topical approaches may have the potential advantage of local effect for locoregional symptoms, but these effects only last for a short time [21,36]. The efficacy seems different for neuropathic pain following breast cancer, where the reported results seem more stable [37]. Additionally, topical agents may be systemically absorbed by the ulcerated mucosa.

Out of the 432 articles which emerged through the database search, only 10 publications [26,27,28,29,30,31,32,33,34,35] met the inclusion criteria: 7 investigated the effects of anticonvulsants on NP [26,27,28,29,31,32,34], 6 of gabapentin [26,27,28,31,32,34], and 1 of pregabalin [29]. The remaining studies investigated the effects of nortriptyline [33], methadone [30], and ketamine [35].

Gabapentin, originally an anticonvulsant, is recommended for the treatment of several neuropathic pain conditions [38]. Pain reduction was found in five of the six studies [27,31,32,33,34] that studied the effects of gabapentin, suggesting that gabapentin could be a positive treatment for NP in HNC patients under RT. However, three of these studies had a high risk of bias on several domains of the Cochrane Collaboration tool [31,32,34]. Moreover, two of them did not have a control group [31,32]. Only the study of Smith et al. [27] had a low risk of bias, providing reliable results. Herman et al. [26] performed a trial to compare high dose gabapentin (2700 mg/d) to lower dosage (900 mg/d). They did not establish a significant difference on pain reduction. In this study, one domain of the Cochrane Collaboration tool revealed a high risk of bias. Kataoka et al. [28] also did not establish a significant difference in pain assessment between the gabapentin and the control group. Here, two domains of the Cochrane Collaboration tool had an unclear risk of bias, and two domains had a high risk of bias.

Pregabalin is also recommended for several chronic neuropathic pain conditions [37]. Jiang et al. [29] showed that pregabalin compared to the placebo demonstrated a significant decrease in pain intensity and severity. This study had a low risk of bias on all domains of the Cochrane Collaboration tool contributing to reliable results as it was a study of high quality.

Nortriptyline, a tricyclic antidepressant, has been found to have analgesic properties and is used for management of NP, but the study included in this review did not demonstrate a better pain relief compared to opioids [33]. Nortriptyline showed sufficient pain control only in some HNC patients. Three domains of the Cochrane Collaboration tool had an unclear risk of bias.

Methadone is a strong opioid also used for the treatment of NP as it has an action on the N-methyl D-aspartate (NMDA) receptor [39]. Haumann et al. [30] found a higher reduction in NRS in the methadone group compared to the one using fentanyl. In their study, three domains of the Cochrane Collaboration tool had a high risk of bias.

Ketamine, a NMDA receptor antagonist, was suggested as an adjuvant in acute pain treatment [39]. Moreover, it is useful in chronic pain patients [40] and beneficial for NP [41]. In the study included in this review, oral ketamine was evaluated as an adjuvant to oral morphine in cancer patients [35]. Even though the study was performed in a mixed population of different cancer sites, we decided to include it as the HNC population results were well described. All four HNC patients had a successful response to oral ketamine. Four domains of the Cochrane Collaboration tool had an unclear risk of bias.

Our review aimed to provide a therapeutic approach for this complex pain entity. A useful algorithm for a pain clinician would be to trial a combination of an opioid with an anticonvulsant/tricyclic antidepressant agent or/and NMDA antagonist (either ketamine or action embedded within the opioid itself—methadone). A previous review by Lefebvre et al. [42] also approached this important problem. Similar to our results, the authors proposed anticonvulsants and nortriptyline, and focused on topical treatments such as doxepin rinse, botulinum toxin, and polymer film containing tetracaine. However, the important role of the NMDA receptor antagonists is missing.

One of the strengths of this study is that it includes the most recent publications. The database search also identified a randomized study of the team of McMenamin where the authors investigated the role of pregabalin [43], 300 mg once/day compared to the placebo, in pain management in HNC patients under chemoradiation. Even though the study is referred as “completed” and “with results”, to the best of our knowledge, the results are not yet published so we were not able to include it in our review.

This systematic review has some limitations. The literature search was not performed in all existing databases. Therefore, it is possible that some studies that support a range of potentially important pain control measures may be missed. For the 10 studies in this review, a relatively small number of patients (506 in total) contributed data creating low statistical power to detect the real effects. Moreover, it was not possible to perform a meta-analysis because of the heterogeneity of the parameters reported in the studies: different pain evaluation tools, and different time points of therapies and pain assessment. A further limitation was the relatively low quality of most of the studies included, which would not make the generalizability of the findings possible.

Based on the results of the examined studies, it is not possible to provide specific recommendations. Further studies, using better standardized parameters (pain assessment, consistent time points for the administration of analgesics, and analgesia evaluation), and new potential therapies for neuropathic pain [44] are necessary. This would help to investigate the effectiveness of NP medication in HNC patients receiving RT. The comparison of different studies investigating the efficacy and safety of the same drugs would further contribute evidence for or against the use of these therapies.

## 5. Conclusions

The prompt diagnosis and treatment of NP is an important and largely unmet medical need in the HNC patient population. While there is a plethora of possible pharmacologic treatments available, only a few studies have been conducted regarding the management of therapy-related NP in HNC patients. Gabapentin, pregabalin, methadone, and ketamine were found to provide some analgesia, with good evidence only in two studies on gabapentin and one on pregabalin. Future studies looking into different pharmacological approaches in patients with HNC treated with RT with NP are necessary as patients should receive the most effective and tolerable treatment for their individual needs.

## Figures and Tables

**Table 1:** Summary of the analyzed studies.

Author	Study Type	Drug	Comparison	Sample Size (Drug/Comparison)	Cancer Site	Radiotherapy
Herman et al., 2020 [26]	PRPS	Gabapentin (2700 mg/d) + hydrocodone and/or paracetamol progressing to fentanyl	Gabapentin (900 mg/d) + methadone	60 (31/29)	Nasopharyngeal, oral, oropharyngeal, laryngeal, hypopharyngeal, unknown primary	IMRT
Smith et al., 2020 [27]	PRPS	Gabapentin + NSAIDs and opioids	NSAIDs and opioids	79 (41/38)	Paranasal sinus, oral, oropharyngeal, nasopharyngeal, hypopharyngeal	NA
Kataoka et al., 2016 [28]	OLPRS	Gabapentin + paracetamol + opioids	Paracetamol + opioids	22 (11/9)	Oral, oropharyngeal, nasopharyngeal, laryngeal, hypopharyngeal	NA
Starmer et al., 2014 [34]	HCS	Gabapentin ± oxycodone	Opioids	46 (23/23)	Tongue base, tonsil, soft palate	IMRT
a. Bar et al., 2010 [31]	RCS	Gabapentin	No	30	Oral, oropharyngeal, thyroid, salivary, skin, unknown primary, laryngeal, paragangliomas	IMRT
b. Bar et al., 2010 [32]	RCS	Gabapentin	No	42	Paranasal sinus, oral, oropharyngeal, laryngeal hypopharyngeal, sino-nasal	IMRT
Jiang et al., 2018 [29]	RCT	Pregabalin	Placebo	128 (64/64)	Oral, oropharyngeal, lip, nasopharyngeal, laryngeal, paranasal sinus	NA
Ehrnrooth et al., 2001 [33]	RaT	Nortriptyline	Morphine (p.o.)	43 (21/22)	Oral, laryngeal, pharyngeal	NA
Haumann et al., 2016 [30]	RCT	Methadone	Fentanyl	52 (26/26)	Not reported	NA
Kannan et al., 2002 [35]	UCOLS	Ketamine (p.o.) + antidepressant and/or anticonvulsant	Antidepressant and/or anticonvulsant	10 (4 HNC pts)	Laryngeal, tongue	NA

HCS: Historically Controlled Study, IMRT: Intensity Modulated Radiotherapy, NA: Not Applied, OLPRS: Open Label Prospective Randomized Study, PRPS: Prospective Randomized Pilot Study, RaT: Randomized Trial, RCS: Retrospective Cohort Study, RCT: Randomized Control Trial, SPC: Standard Pain Control, UCOLS: Uncontrolled Open-Label Study.

**Table 2:** Used tools and timing to evaluate the analgesic efficacy of the used therapy.

Author	Drug	Comparison	Sample Size (Drug/Comparison)	Pain Assessment	Timing
Herman et al., 2020 [26]	Gabapentin (2700 mg/d) + hydrocodone ± paracetamol progressing to fentanyl	Gabapentin (900 mg/d) + methadone	60 (31/29)	OMWQ-HN, EORTC QLQ-H&N35	Day 1
Smith et al., 2020 [27]	Gabapentin + NSAIDs and opioids	NSAIDs and opioids	79 (41/38)	VHNSSv2	Day 1
Kataoka et al., 2016 [28]	Gabapentin + paracetamol + opioids	Paracetamol + opioids	22 (11/11)	VAS	Day 1
Starmer et al., 2014 [34]	Gabapentin ± oxycodone	Opioids	46 (23/23)	absent (0), mild (1), moderate (2), severe (4)	Week 1
a. Bar et al., 2010 [31]	Gabapentin ± opioids	no	30	Opioid use	Week 2
b. Bar et al., 2010 [32]	Gabapentin ± opioids	no	42	Opioid use	Week 2
Jiang et al., 2018 [29]	Pregabalin	Placebo	128 (64/64)	NRS, LANSSQ, BPI-SF	After RT
Ehrnrooth et al., 2001 [33]	Nortriptyline	Morphine (p.o.)	43 (21/22)	VAS, McGill PQ, Likert scale	After insufficient pain management with weak analgesics (paracetamol)
Haumann et al., 2016 [30]	Methadone	Fentanyl 12 mg/h (patch)	52 (26/26)	NRS, DN4, BPI	When pts reported NRS ≥ 4 and DN4 ≥ 4
Kannan et al., 2002 [35]	Ketamine (p.o.) + antidepressant and/or anticonvulsant	Antidepressant and/or anticonvulsant	4	NRS	NRS > 6, neuropathic pain based on clinical criteria

OMWQ-HN: Oral Mucositis Weekly Questionnaire adapted for Head and Neck Cancer; EORTC QLQ-H&N35: European Organization for Research and Treatment of Cancer Quality of Life Questionnaire—Head and Neck Module; LANSSQ: Leeds Assessment of Neuropathic Symptoms and Signs questionnaire; VHNSSv2: Vanderbilt Head and Neck Symptom Severity Survey version 2.0; NRS: Numeric Rating Scale; VAS: Visual Analog Scale; BPI-SF: Brief Pain Inventory; McGill PQ: McGill Pain Questionnaire; DN4: Douleur Neuropathique 4; p.o.: per os.

**Table 3:** Efficacy and safety of the used therapies.

Author	Drug/Comparison	Sample Size (Drug/Comparison)	Indication	Drug Dose	Outcome	Adverse Effects
Herman et al., 2020 [26]	Gabapentin (2700 mg/d) + hydrocodone and/or paracetamol progressing to fentanyl/Gabapentin (900 mg/d) + methadone	60 (31/29)	Pain during therapy	2700 mg/d (p.o.)	No significant difference *p* = 0.87	3% of pts discontinued treatment due to intolerance to gabapentin
Smith et al., 2020 [27]	Gabapentin + NSAIDs and opioids/NSAIDs and opioids	79 (41/38)	Pain during therapy	2700 mg/d (max, p.o.)	Pain reduction *p* = 0.004	Fatigue and sedation
Kataoka et al., 2016 [28]	Gabapentin + paracetamol + opioids/Paracetamol + opioids	22 (11/11)	Pain during therapy	900 mg/d (p.o.)	No significant difference *p* = 0.552	Somnolence, allergic skin reaction
Starmer et al., 2014 [34]	Gabapentin ± oxycodone/Opioids	46 (23/23)	Pain during therapy	2700 mg/d (p.o.)	Pain reduction *p* = 0.0003	Vertigo, headaches, fatigue
a. Bar et al., 2010 [31]	Gabapentin ± opioids/no	30	Pain during therapy	2700 mg/d (median, p.o.)	Wk 1 and 2: 86% of pts required no pain medication.	Dizziness, nausea, vomiting, skin rash
					Wk 3 and 4: 10% of pts required low doses of opioids (15–30 mg/day of roxicodone).	
					Wk 5 and 6: 35% of pts required low doses of opioids (15–40 mg/day of roxicodone)	
b. Bar et al., 2010 [32]	Gabapentin ± opioids/no	42	Pain during therapy	2700 mg/d (median, p.o.)	Wk 2: 12% of pts required median dose of 10 mg/day of oxycodone-equivalent.	Dizziness
					Wk 3: 33% of pts required median dose of 10 mg/day of oxycodone-equivalent.	
					Wk 4: 55% of pts required median dose of 30 mg/day of oxycodone-equivalent.	
					Wk 5 and 6: 71% of pts required median dose of 60 mg/day of oxycodone-equivalent.	
Jiang et al., 2018 [29]	Pregabalin/Placebo	128 (64/64)	Neuropathic pain	600 mg/d (max, p.o.)	Pain reduction *p* = 0.003	Dizziness, somnolence, headache, diarrhea, peripheral edema
Ehrnrooth et al., 2001 [33]	Nortriptyline/Morphine (p.o.)	43 (21/22)	Pain during therapy	150 mg/d (max, p.o.)	Significantly higher VAS scores at 1 and 2 wk after randomization in nortriptyline group compared to morphine group (*p* = 0.007 and 0.04, respectively)	Nausea, vomiting
Haumann et al., 2016 [30]	Methadone/Fentanyl	52 (26/26)	Neuropathic pain	5 mg/d (p.o.)	Significantly higher reduction at 1 and 3 wk in NRS scores in methadone group compared to fentanyl group (*p* = 0.011 and 0.03, respectively)	Dry mouth, sleepiness, dizziness, nausea, vomiting, constipation
Kannan et al., 2002 [35]	Ketamine (p.o.) + antidepressant and/or anticonvulsant	4	Neuropathic pain	1.5 mg/kg/d (p.o.)	Pain reduction after adding ketamine (*p* = 0.12)	Nausea, drowsiness, anorexia, vomiting, tiredness

VAS: Visual Analog Scale; NRS: Numeric Rating Scale; p.o.: per os; wk: week.

**Table 4:** Results of the “vote-counting”.

Author, Year, Type of Study	Country	Drug	Negative Effect	Zero Effect	Positive Effect
Herman et al., 2020, [26] RPPS	USA	Gabapentin		✓	
Smith et al., 2020, [27] RPPS	USA	Gabapentin			✓
Kataoka et al., 2016, [28] RPPS	Japan	Gabapentin		✓	
Starmer et al., 2014, [34] HCS	USA	Gabapentin			✓
Bar et al., 2010, [31] RCS	USA	Gabapentin			✓
Bar et al., 2010, [32] RCS	USA	Gabapentin			✓
Jiang et al., 2018, [29] RCT	China	Pregabalin			✓
Ehrnrooth et al., 2001, [33] RaT	Denmark	Nortriptyline		✓	✓
Haumann et al., 2016, [30] RCT	Netherlands	Methadone			✓
Kannan et al., 2002, [35] UCOLS	India	Ketamin			✓
TOTAL			0	3	7

RCT: Randomized Control Trial, RCS: Retrospective Cohort Study, RaT: Randomized Trial, SPC: Standard Pain Control, HCS: Historically Controlled Study, PRPS: Prospective Randomized Pilot Study, UCOLS: Uncontrolled Open-Label Study.

## Data Availability

All the data reported in this study would be available by the corresponding author, on reasonable request.
